# Investigating the population health impact of an oral tobacco–derived nicotine pouch product utilizing a three-product tobacco use population model

**DOI:** 10.3389/fpubh.2026.1772045

**Published:** 2026-02-25

**Authors:** Raheema Muhammad-Kah, Thaddaeus Hannel, Lai Wei, Hui Cheng, Sucharitha Iyer, Mohamadi Sarkar

**Affiliations:** Center for Research and Technology, Altria Client Services LLC, Richmond, VA, United States

**Keywords:** agent-based modeling, mortality estimates, nicotine pouches, oral tobacco-derived nicotine, population health, three-product population modeling

## Abstract

**Background:**

This study employed a three-product agent-based model (ABM) to evaluate the potential population-level effects of granting market authorization for *on!*^®^ nicotine pouches, an oral tobacco-derived nicotine product. Previous research has typically relied on simplified two-product models comparing cigarette use in a “Base Case” with a “Modified Case” scenario, limiting the ability to reflect real-world multi-product tobacco use patterns. To address this, the ABM in this study incorporated cigarettes and smokeless tobacco (ST) products in the Base Case and added the *on!*^®^ nicotine pouches as the Test Product in the Modified Case.

**Methods:**

Developed in MATLAB^®^ version 9.2, the model consisted of a transition sub-model, which simulated annual changes in product use based on national survey data and Test Product-specific studies, and a mortality sub-model, which linked survival outcomes to product transitions using an excess relative risk estimate of 5% for the Test Product relative to cigarettes. The simulation ran over an 80-year time horizon, predicting individual-level agent dynamics and annual transitions.

**Results:**

Model outputs indicated that introducing the Test Product could prevent approximately 476,000 premature deaths, reduce cigarette prevalence by 0.6 percentage points, and ST product use by 0.3 percentage points, while increasing Test Product use by 1.6 percentage points.

**Conclusion:**

These findings suggest that regulatory authorization of *on!*^®^ nicotine pouches could yield a net public health benefit by lowering all-cause mortality and reducing the prevalence of use of tobacco products associated with greater health risks.

## Introduction

Computational models have been employed to evaluate the population-level effects of introducing new tobacco products with varying risk profiles to assess the net impact on public health. Studies utilizing these models consistently indicate that transitioning from traditional cigarettes to alternative products such as e-cigarettes, nicotine pouches, or heated tobacco products (HTPs) yields a net positive impact on public health. This conclusion is based on observed reductions in smoking prevalence, as well as declines in tobacco-related mortality and morbidity across the population (1–7). Computational models have also become an increasingly valuable tool in shaping public health policy decisions in the tobacco regulatory space (8–10).

Various types of computational models have been employed to estimate the impact on population health. For example, systems dynamic modeling has quantified all-cause mortality based on estimated lifetime tobacco exposure for hypothetical cohorts (11) and explored the public health implications of potential regulations mandating low-nicotine cigarettes (8, 10).

We employed an Agent-Based Modeling (ABM) approach to simulate how individual “agents” interact with their environment based on predefined, real-world parameters, enabling predictions of population-level outcomes (12, 13). In this framework, each agent functions autonomously, making decisions and shaping its own trajectory in response to specific stimuli (12). By representing a diverse cross-section of the population, ABMs capture the heterogeneity and complexity of real-world dynamics, including temporal and demographic shifts driven by births, deaths, immigration, and other factors influencing tobacco use patterns, transitions, survival, and mortality (13). The dynamic nature of ABMs allows them to incorporate uncertainty and generate insights at both population and subgroup levels, making them a more robust and flexible alternative to static compartmental models for assessing the public health impacts of tobacco products. ABM has been applied to evaluate the impact of various tobacco control measures, including smoking cessation programs, smoke-free policies, and the effects of emerging products such as electronic cigarettes on smoking behaviors within social networks and overall prevalence (14–16).

Most published models use a two-product framework: a Base Case that includes only cigarettes, and a Modified Case that adds a new tobacco product to assess its impact in a market where cigarette use predominates at the population level. However, this approach may not fully reflect real-world conditions. For example, in the United States (U. S.), both cigarettes and smokeless tobacco (ST) products historically represent commonly used forms of tobacco use. Earlier three-product computational modeling frameworks—designed to evaluate the public health impacts of heated tobacco products (HTPs) and e-cigarette market authorization in global markets—were constrained by the limited availability of large-scale, publicly accessible datasets on tobacco use histories and product transition patterns (5, 6).

We present the ALCS ABM,^1^ which we previously validated and described (4, 17). Our three-product model integrated data from multiple verified public sources to enhance demographic and tobacco transition analyses while minimizing assumptions. The ALCS ABM design parameters complied with best practices described for mathematical modeling optimized for public policy and health-care disciplines (18).

The ALCS ABM was developed using MATLAB^®^ version 9.2 and validated to simulate population health impacts from 2020 to 2,100. The model compares two scenarios: a “Base Case,” representing the current landscape with cigarette and ST product use, and a “Modified Case,” which incorporates the market authorization of *on!*^®^ nicotine pouches^2^ alongside cigarettes and ST products. Using prevalence rates based on current usage and mortality endpoints, the model evaluates future population health outcomes, including scenarios where users consume more than two tobacco products following regulatory authorization of *on!* nicotine pouches in the U. S.

## Materials and methods

### Model framework for estimating the impact of market authorization of *on!* nicotine pouches

**Figure 1:**
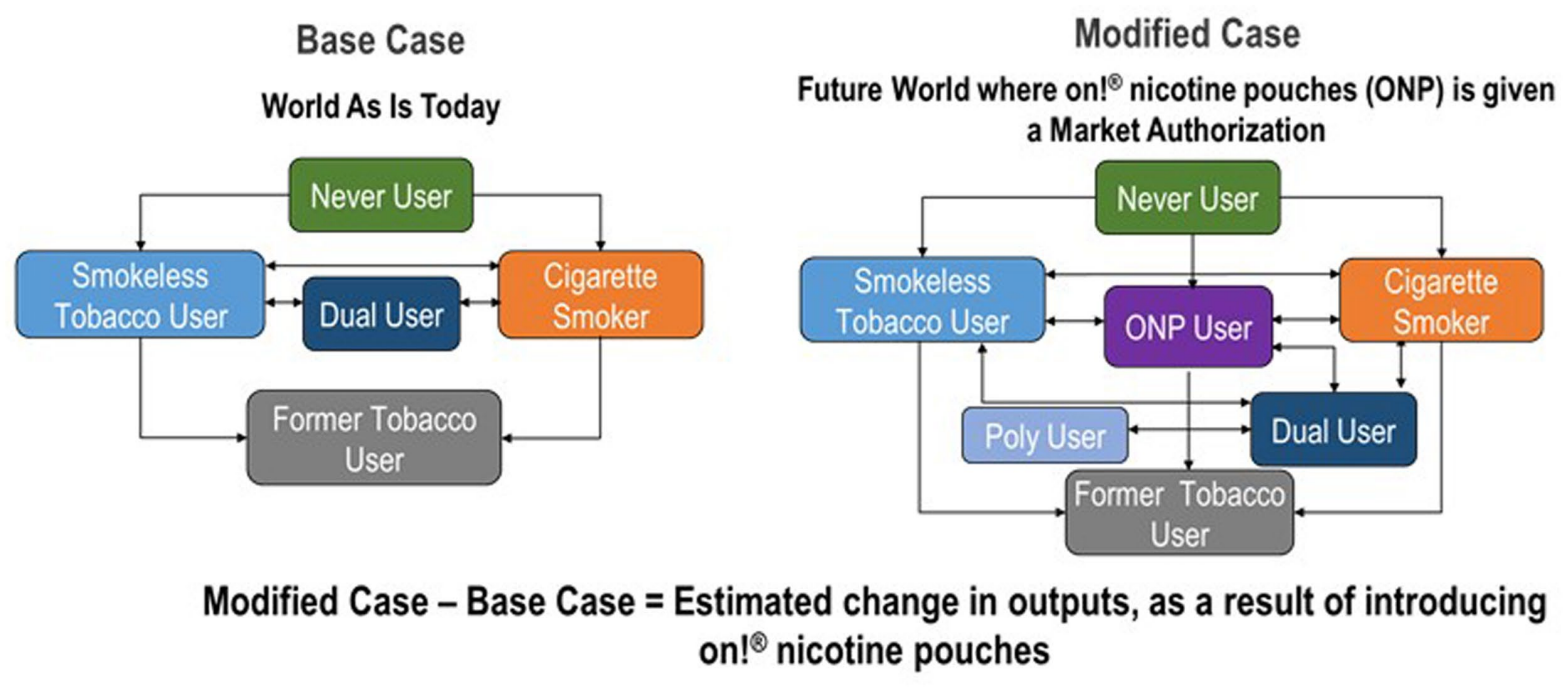
Conceptual framework for modeling the impact of on! nicotine pouch product authorization.

The framework presented estimated the overall health impact of market authorization of *on!* nicotine pouches by comparing the difference in tobacco use prevalence and all-cause mortality between a Base Case and Modified Case over an 80-year period. This duration was selected to provide adequate time to predict the long-term impact on mortality (See Figure 1).

### Base case scenario

The Base Case, or status quo scenario, took into consideration the current U. S. population, inclusive of those who use both cigarettes and ST products. The transition probabilities for the Base Case, consisting of exclusive users of cigarettes and ST products, and dual users of both tobacco products, were obtained from nationally representative data and from peer-reviewed literature.

### Modified case scenario

The Modified Case scenario reflected a future state in which we assessed potential changes to the Base Case due to market authorization of *on!* nicotine pouches, resulting in shifts in the prevalence of both cigarette and ST product consumption. This study modeled the *on!* nicotine pouches introduction in a scenario of prevalent cigarette and ST product use, and thereby differentiates from prior work that determined new product introduction in the context of only cigarette use (3, 7, 10, 11, 19–21). Transitions related to nicotine pouches were estimated from behavioral studies and nationally representative data.

### Model overview

**Figure 2:**
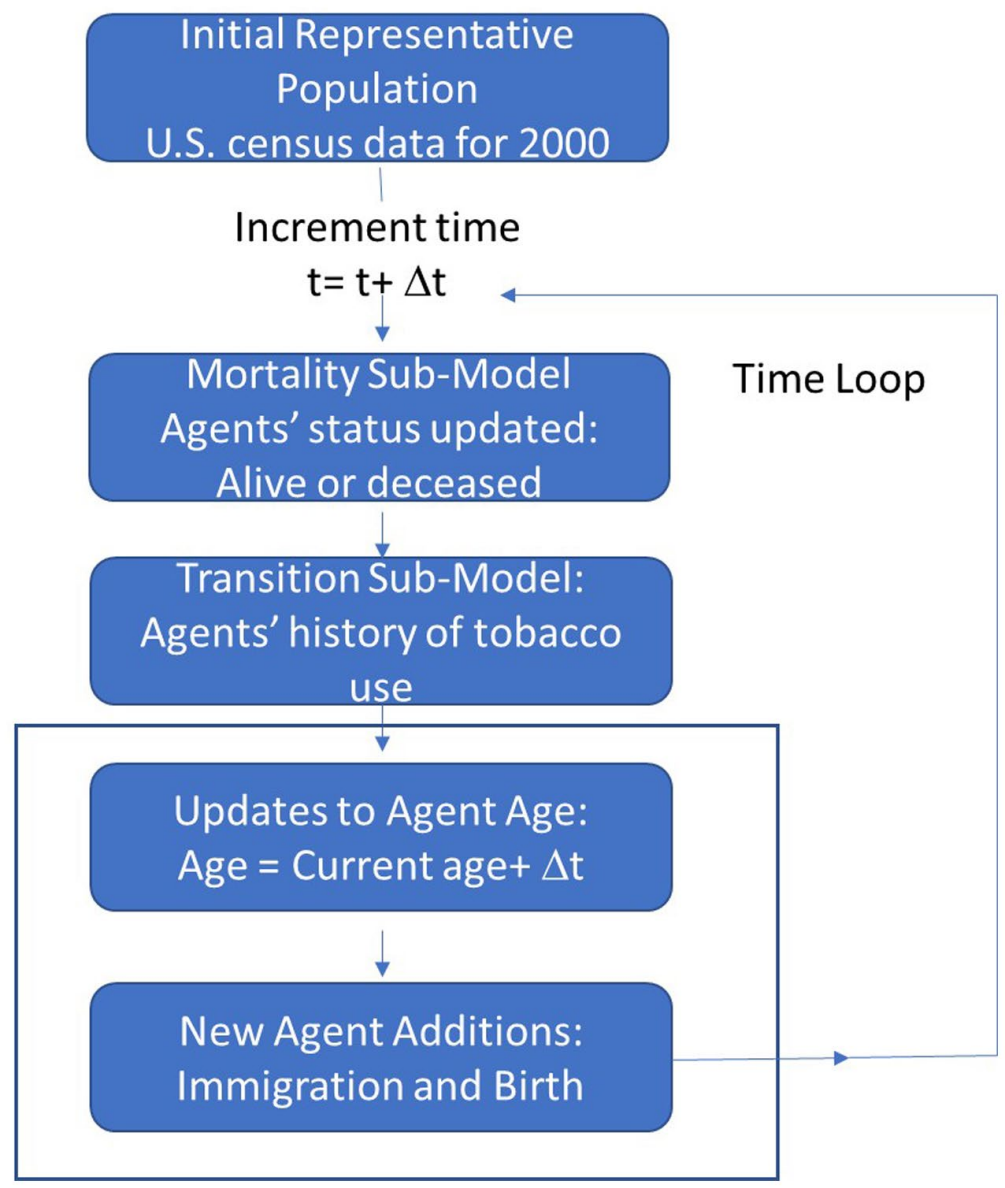
Model overview.

The ALCS ABM involved the following four steps. First, an initial population was generated, then at each 1-year time step, the survivability of agents was evaluated based on mortality risk factors dependent on their tobacco use history (i.e., Mortality Sub-Model). Agents who survived transitioned between tobacco use states (i.e., Transition Sub-Model), and finally, new agents were introduced through birth and immigration on an annual basis (See Figure 2).

### Generating the starting population

A representation of the U. S. population for the year 2000 in terms of age, sex, and tobacco use status was initialized with a hypothetical population of 2.81 million agents (1/100th of the year 2000 U. S. population). To simulate the entire U. S. population, the model was iterated 100 times. The initial population mirrored the age and sex distribution data from the U. S. Census for the year 2000. Each agent in the initial population was assigned one of six tobacco use statuses representative of people who have never smoked (NT), people who currently smoke cigarettes (CS), people who previously smoked cigarettes (FS), people who currently use smokeless tobacco products (ST), people who previously used smokeless tobacco products (FST), and people who use both cigarettes and ST products (DU1). Tobacco use status was assigned by sex and ages ≥ 18 using information from the National Health Interview Survey (NHIS) Sample Adult Questionnaire data for the year 2000 (22). In our analysis of NHIS data, CS were defined as those who reported having smoked at least 100 cigarettes in their lifetime and currently smoking every day or some days. FS were defined as those who reported having smoked at least 100 cigarettes in their lifetime and are currently not smoking at the time of interview. ST were defined as those currently using snuff or chewing tobacco every day or some days. FST were defined as those who are not at all using snuff or chewing tobacco. NT were defined as those who have never smoked or did not reach the 100-cigarette lifetime threshold. These definitions are commonly used by the Centers for Disease Control and Prevention (CDC) to estimate the prevalence of tobacco use among the U. S. adult population (23).

The NHIS does not provide tobacco use information for ages < 18 years; therefore tobacco use status assigned to the younger U. S. population, ages 10–17, by sex were estimated from the data for the year 2000 from the National Youth Tobacco Survey (NYTS), a nationally representative survey of middle and high school students focused exclusively on patterns of tobacco use. In our analysis of the NYTS, NT were defined as youth who have never smoked or not smoked 100 cigarettes in their lifetime or never having used snuff or chewing tobacco in their lifetime; CS were those who reported having smoked 100 cigarettes in their lifetime and having smoked in past 30 days; FS were those who reported having smoked 100 cigarettes in their lifetime but not having smoked in past 30 days; ST were those who reported having used snuff or chewing tobacco at least 1 day during the past 30 days; and FST were those who reported having not used snuff or chewing tobacco at all in the past 30 days.

Each agent in the initial population is assigned a tobacco use history, which is updated over the 100-year simulation timeframe from 2001 to 2,100. Agents who initialized as CS or FS were assigned with their associated years of smoking or years stopped smoking, respectively, and the age(s) at which the agent initiated and/or stopped smoking. Age and sex-specific probabilities from the U. S. birth cohort smoking history data developed by Jeon et al. (22) were used to assign when a CS or FS in the model’s starting population initiated or stopped smoking. The age- and sex- specific cigarette smoking initiation and cessation probabilities were generated by researchers who used NHIS surveys administered from 1964 to 2015 to estimate birth cohort smoking histories (24). This initiative was part of the Cancer Intervention and Surveillance Modeling Network (CISNET) Lung Working Group, sponsored by the National Cancer Institute. The ALCS ABM does not allow for relapse scenarios. The CISNET cessation rates reflect successful smoking cessation for at least 2 years, differentiating between successful cessation (i.e., cessation for 2 years or more, with relapse being highly unlikely) and short-term cessation followed by relapse. ST product initiation for males and females was based on published literature sources (25, 26).

After the initial population was generated, the Mortality and Transition Sub-Models were executed in 1-year time intervals throughout the simulation time frame.

### Mortality sub-model

A mortality sub-model was used to estimate the survival probability of each agent based on their age, sex, and history of current or former tobacco use. The mortality sub-model was developed using data from a Kaiser Permanente (KP) Medical Care Program Cohort study (27), which included number of deaths, smoking the status, age, sex, person-years, and years since quitting smoking. The KP Study data were adjusted using the Human Mortality Database to be representative of the U. S. population in the year 2000. Mortality rates throughout the simulation time frame were further adjusted to account for expected age-specific changes in mortality over time using the methodology described by Carter et al. (28). Mortality rates associated with the use of ST products and nicotine pouches were estimated based on the excess relative risk (ERR) of these products relative to smoking. The ERR quantifies the risk posed by using a new product relative to the risk of using a reference product, such as a cigarette, traditionally estimated based on epidemiological studies (29). An ERR of 0.09 (or 9% the risk of smoking) was assigned for ST product use, as previously estimated for current adults who use ST products relative to adults who smoke combustible cigarettes (17). This estimated ERR is consistent with the relative risk hierarchy derived from the combination of lifetime cancer risk and epidemiological analysis of U. S. ST products (30). We based the ERR on all-cause mortality hazard ratios for ST product use and cigarette smoking from two large, nationally representative linked mortality datasets (31).

An ERR of 0.05 (or 5% the risk of smoking) was assigned for nicotine pouches. Given that nicotine pouches have not been on the market for an extended period of time, no long-term epidemiological data specific to this category of products currently exists to estimate their ERR. Therefore, we considered the outcomes of a Multi-Criteria Decision Analysis model (MCDA approach) developed by an international expert panel (32). The panel assigned the relative importance of different types of tobacco product harm and reported an ERR of 5% for e-cigarettes and 2% for oral nicotine-containing products [e.g., nicotine replacement therapies (NRTs)]. In a more recent publication, Murkett et al. (30) assessed the risks of tobacco products, including NRTs and nicotine pouches, relative to cigarette smoking. NRTs were estimated to have a relative risk of 0.4% while nicotine pouches were estimated to be 0.1%.

The ERR for users of two or more than two tobacco products was set to be the same as the highest relative risk among the tobacco product use combination(s), consistent with an approach adopted by other researchers (8, 10). For example, for users of two or more than two tobacco products of nicotine pouches with cigarettes, we assigned the same risk as cigarette smoking.

### Transition sub-model

**Figure 3:**
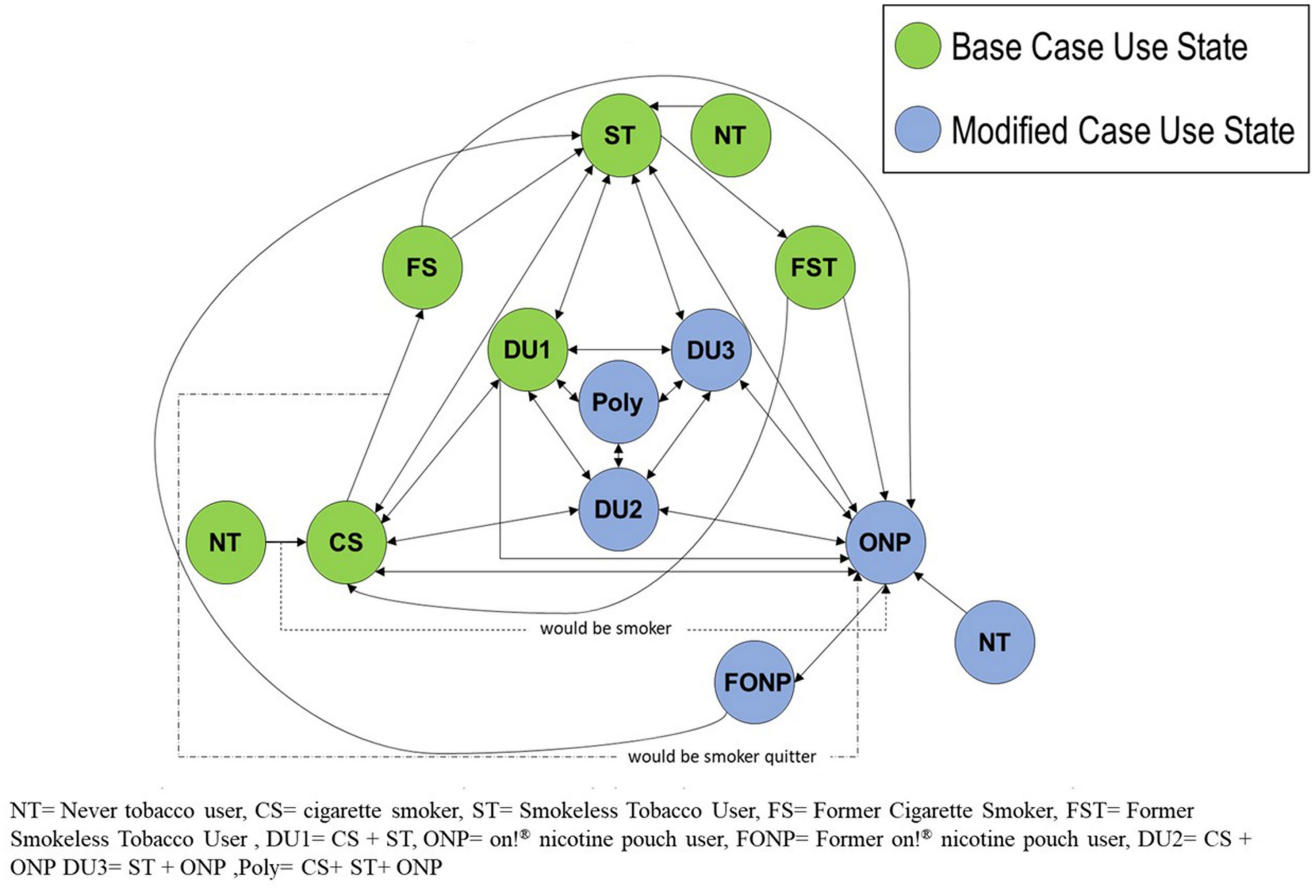
Modeling framework.

At each time interval within a simulation, agents were provided with an option to change or maintain their current tobacco use status. These decisions were governed by the agent’s defined current status, age, and sex-specific transition probabilities. See Figure 3 for the ALCS ABM transition diagram for the Base Case and Modified use states.

### Transition probabilities for the base case scenario and sources

The Base Case scenario took into consideration the current U. S. population consisting of adults who use both cigarettes and ST products. Transition probabilities for cigarette initiation and cessation by sex and age were obtained from CISNET, which reports the smoking history of U. S. birth cohorts using NHIS data (22, 23). As previously mentioned, at least 2 years of successful cessation rates were estimated by CISNET; therefore, relapse was not considered in the ALCS ABM scenarios. Steep declines observed in smoking prevalence between 2014 and 2018 were not reflected in CISNET cessation rates, which only cover the time period between 2000 and 2013. Therefore, from 2014 onwards, the data analyses of PATH Waves 1 through 4 informed the ALCS ABM cessation rates. The cessation rate calculation methodology in PATH analyses was considered congruent with CISNET stipulated timeframe criteria for cessation, wherein cigarette users in Wave 1 followed through to Wave 4, became adults who previously smoked (FS) in Wave 2, and retained this tobacco use status through Wave 4.

Transition probabilities between users of cigarettes and ST products were estimated from PATH Waves 1 and 2 data among adults who never used snus, given the longitudinal and nationally representative nature of the surveys, along with published literature sources (25, 26). These transitions were estimated among adults who never used snus in the Base Case since snus was used as a proxy for nicotine pouches in assessing the Modified Case transitions obtained from PATH Waves 1 and 2. At the time of this analysis, nicotine pouch data was not available in PATH and we believed snus to be a reasonable proxy. In the absence of reliable transition data for *on!* nicotine pouches, we used snus estimates from PATH for three reasons—first, both snus and the candidate products are pouched products; second, both products are intended for oral use, to be placed under the upper lip; and third, they are both spit-free products.

### Transition probabilities for the modified case scenario and sources

The Modified Case reflected a future state of changes in transitions between tobacco product usage due to the introduction of *on!* nicotine pouches in the U. S. population, where cigarette smoking and ST product use already exist. We estimated transition probabilities for *on!* nicotine pouches from two behavioral studies a Perception and Behavioral Intentions Study (PBI Study) and Actual Use Study (AUS) which we further adjusted to reflect the U. S. population.

The PBI Study was used to estimate the transition probabilities of *on!* nicotine pouches and consisted of both adults who use tobacco and adults who do not use tobacco products. The study was a quantitative, pre-post monadic, self-administered online survey, conducted nationwide (based on quotas from 2018 NHIS) using multiple recruitment models. The study samples consisted of 4,646 adults participants. Participants were assigned one of six subgroups based on their tobacco use: (i) adults who smoked that were planning to quit (ASPQ), (ii) adults who smoked that were not planning to quit, (iii) adults who dual used both cigarettes and ST products, (iv) adults who used ST products, (v) adults who previously used tobacco and (vi) adults who never used tobacco, and represented a variety of current tobacco use products. The PBI Study sought to determine the effects of *on!* nicotine pouch promotional material exposure on changes in behavioral intentions to try, use, use two tobacco products, switch, or quit smoking or quit all tobacco use. The following outcomes were assessed: intention to try, likelihood to try, intention to use, intention to quit smoking, and intention to purchase candidate products.

Observations from adults who never used tobacco products and adults who previously used tobacco product groups from the PBI Study were used to inform the inputs for initiation and re-initiation among non-users, as summarized in below Table 1. The final model inputs resulted from the PBI Study estimates obtained from the measure likely to use *on!* nicotine pouches and PATH estimate of the measure of snus onset among those who were susceptible to using snus. For example, the initiation of *on!* nicotine pouches were estimated to be 0.02%, obtained by multiplying PBI Study outputs with PATH output measures (i.e., 3.6% × 0.5).

**Table 1:** Estimations of model inputs for nonuser annual transition rates.

Transition	Input for estimate of transition rates		Estimated transition rate (model input)
	PBIS What is the proportion of nonusers who are likely to use candidate products? Measure: Likelihood to Use^a^	PATH Among nonusers who are likely to use candidate products, what is the proportion who will actually use the candidate products? (We use snus as proxy) Measure: Onset of snus use^b^	
NT → OTDN (initiation)	3.6%	0.5%	0.02%
FS → OTDN (re-initiation)	10.7%	1.6%	0.17%
FST → OTDN (re-initiation)	12.5%	2.1%	0.26%

^a^Likelihood to use on! Was defined as the proportion of participants having a likelihood based on the intention score at or above 3.5 and who responded “Yes” to the purchase intent question. ^b^Onset of snus use was defined as using snus for the first time between Wave 3 and Wave 4 assessment. Susceptible to snus use was defined as any non-definitive-negative an answer (including “definitely yes,” “probably yes,” and “probably no”) to the question “Do you think you will use snus in the next year?” at Wave 3 assessment.

Two additional subpopulations that may potentially change their behaviors as a result of the authorization of *on!* nicotine pouches were classified as the “would-be smoker” and the “would-be smoking quitter.” We used data from the PBI Study and applied adjustments based on PATH to estimate the proportion of “would-be smokers.”

Would-be smokers were defined as adults who never used tobacco who would have otherwise started smoking cigarettes but used *on!* nicotine pouches instead and could potentially be considered as a pathway for intercepting smokers. The proportions of “would-be smokers” were computed using the following steps:

1) We first estimated the proportion of respondents in the adults who never used tobacco group (2.4%) from the PBI Study who reported an intention to use *on!* nicotine pouches with a score equal to, or greater than, 3.5 after exposure to the promotional materials and answered, “somewhat agree,” “agree,” or “strongly agree” to the question about planning to smoke in the next 30 days.
2) Next, we applied the estimate for initiation of *on!* nicotine pouches (0.02%) as shown in Table 1. We used the product of the two estimates to calculate the proportion of “would-be smokers” (2.4% × 0.02% = 0.0005%). In the model, this proportion was deducted from the cigarette initiation rate to represent the proportion of new adults who smoke who were potentially intercepted by the authorization of *on!* nicotine pouches.

“Would-be smoking quitters” were defined as adults who smoke cigarettes who would have otherwise quit cigarette smoking but switch to *on!* nicotine pouches instead of quitting cigarette smoking. This transition could be viewed as potentially intercepting likely cessation. The proportion of “would-be smoking quitters” were computed using the following steps:

1) First, we estimated the proportion of adults who smoke who were planning to quit cigarette smoking pre-exposure to *on!* promotional materials and subsequently changed to not planning to quit post-exposure in the adults who smoke planning to quit (ASPQ) group in the PBI Study (8.8%).
2) Second, we estimated the proportion (14.0%) of adults who smoke who were planning to quit in the next 30 days using PATH Wave 4 data among established current smokers who had never used snus and were not current established users of ST products. We restricted the population to those who had never used snus and were not currently established users of ST products, to mimic the ASPQ group in the PBI Study.
3) Finally, we used PATH to estimate the proportion of sustained cessation among Wave 1 currently established adults who smoke (11.0%). We used the product of the above three estimates to simulate the proportion of “would-be quitters” (8.8% × 14.0% × 11.0% = 0.14%).

A six-week *ad libitum* AUS was used to estimate changes in transition probabilities between *on!* nicotine pouch and other specific tobacco-use states. This multi-site, observational study where adults who smoke and use ST products expressed intent to use *on!* nicotine pouches completed a trial use period and at the end of the six-week extended use period, responded to a survey detailing their current tobacco use, intentions to use, or not use, *on!* nicotine pouches, appeal of *on!* Nicotine pouches: reasons for use or discontinuation. Nicotine pouches, and intentions to quit smoking (33). The participants in the AUS represented only a subset of the population of adults who smoke cigarettes; therefore, we adjusted the proportions estimated from the AUS based on the PBI Study to estimate transition probabilities representative of the U. S. adult population who smoke cigarettes and use ST products. The participants in the PBI Study were recruited based on quotas from NHIS, a nationally representative survey; we weighted the proportions related to intentions to try and use from the AUS against the PBI Study results. We used the following steps to generate *on!* nicotine pouch population level estimates:

1) *Likelihood to try*: we estimated this proportion from the PBI Study, based on adults who smoke cigarettes with an intention to try ≥3.5 and positive purchase intent.^3^
2) *Likelihood to use*: we estimated this proportion based on the PBI Study, among those who were likely to try (shown in step 1) based on the highest intentions to use (i.e., post-exposure composite score of 6 which translated to “definitely use”) and positive purchase intent.
3) *Likelihood to switch*: we determined the switching transition from the AUS among adults who smoke who were using the candidate product and stopped using conventional cigarettes in the final week of the AUS.

For example, estimated switching rate (adults who smoke cigarettes switching to *on!* nicotine pouches at the population level) were calculated as: Likelihood to try (40.8%) × Likelihood to Use (7%) × Likelihood to Switch (27.6%) = 0.76%.

We performed sensitivity analyses for the transition probability estimates by creating two additional scenarios of lower and upper limits of the candidate product-specific transition probabilities:

- *Lower bound scenario*: we assessed a lower limit using more restrictive transition probabilities. We estimated the switching transition by further including a measure of confirmation of quitting either smoking or ST products based on an end-of-study survey beyond the criteria described in Step 3 for the Modified Case scenario. Lower bound transition probabilities were estimated by following steps 1–3 above and multiplying the proportion of individuals who confirmed completely quitting.
- *Upper bound scenario*: we assessed an upper limit by using less restrictive transition probabilities. In this scenario, we utilized the proportion of AS that had a post-exposure composite score of 5–6 (very likely to, definitely) and positive purchase intent. For example, this less restrictive criterion resulted in a larger proportion (31%) classified as likely to use for the CS → *on!* nicotine pouch transition compared to the 7% for the more restrictive criteria. Upper bound transition probabilities were estimated by following steps 1–3 above.

Table 2 depicts the estimated population-level annual transition rates for *on!* nicotine pouch use for the Modified, Lower, and Upper Bound Scenarios. For all other transitions used in the Modified Case Scenario, we relied on analysis of PATH Wave 1 and Wave 2 data using snus as a proxy for nicotine pouches to estimate remaining transition probabilities related to *on!* nicotine pouches.

**Table 2:** Model inputs for product-specific user annual transition rates for the modified case, lower-, and upper-bound scenarios.

Transition	Lower bound	Modified case	Upper bound
CS → ONP	0.44%	0.76%	3.47%
CS → CS + ONP	1.98%	1.98%	9.01%
ST → ONP	1.16%	2.61%	16.59%
ST → ST + ONP	1.40%	1.40%	6.22%
CS + ST → CS + ST + ONP	1.32%	1.32%	3.86%
CS + ST → CS + ONP	4.04%	4.96%	14.56%
CS + ST → ST + ONP	0.12%	0.21%	0.60%
CS + ST → ONP	1.15%	2.43%	7.12%

CS, cigarette smoker; ST, smokeless tobacco product user; ONP, *on!* nicotine pouch user Modified Case: Likelihood to try × Likelihood to Use × AUS Behavioral Switch Lower Bound: scenario applies the confirmation of quitting either smoking or ST products at the end of study survey in the AUS Upper Bound: scenario uses transition probabilities derived from Likelihood of Use based on moderate to high intentions to use (i.e., post-exposure composite score of 5–6). Likelihood to Try = Intentions to try on! > = 3.5 and Positive Purchase Intent Likelihood to Use = Among those who were likely to try that have the highest intentions to use (i.e., post-exposure composite score equal to 6) and positive purchase intent AUS Behavioral Switch = Proportion of AUS participants that complete the study and make a behavioral switch.

## Results

### Model validation

**Figure 4:**
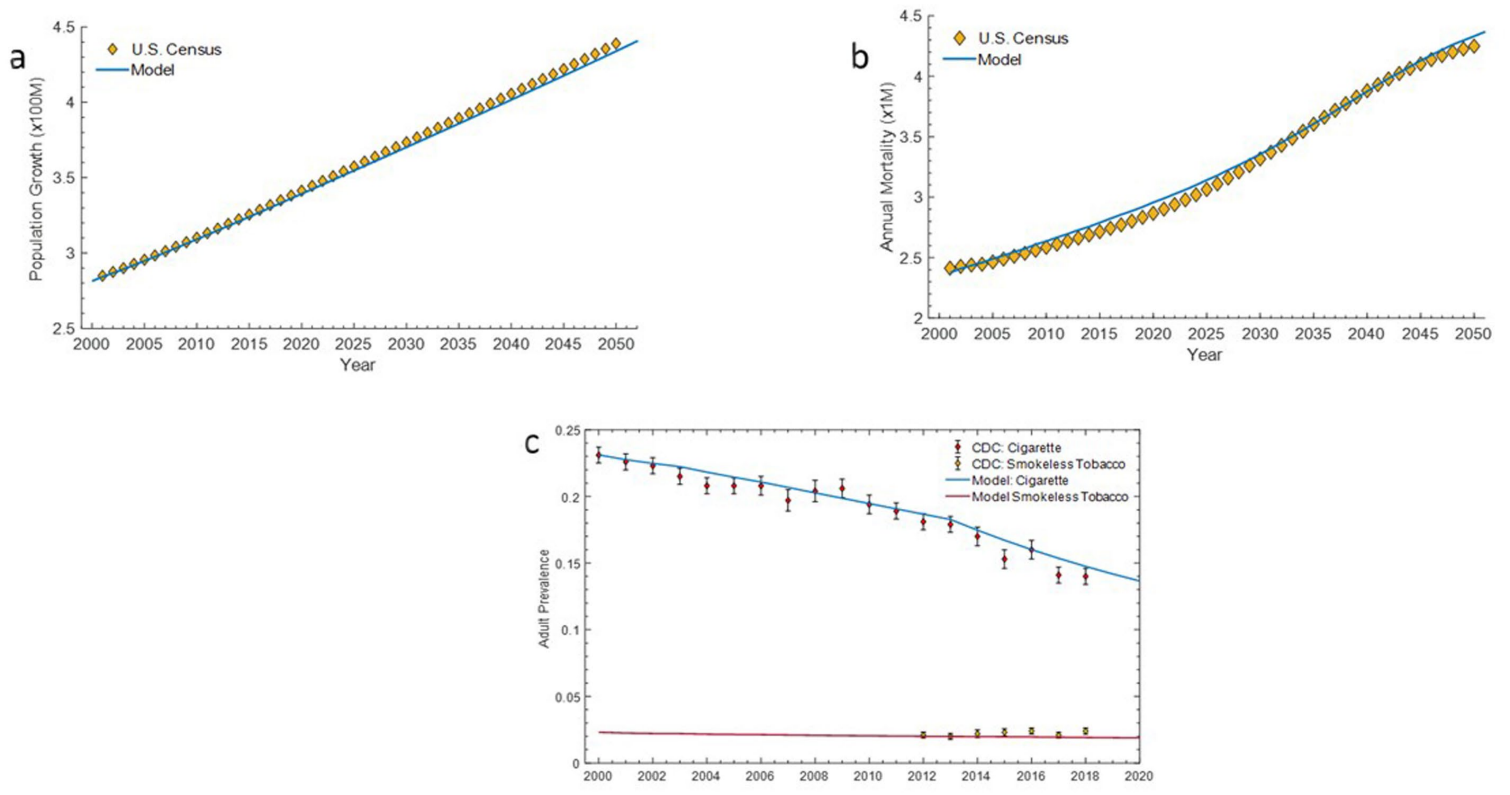
ALCS ABM and U.S. Census Bureau projections for U. S. population growth (4a) and annual mortality (4b). ALCS ABM predictions and CDC reported prevalence of cigarettes and smokeless tobacco products in the U.S. population (4c).

The model results were compared with published population projections from the U. S. Census and prevalence estimates reported by the CDC based on NHIS data (23). The estimated projections for both the U. S. population growth and annual mortality under the Base Case scenario were similar (Figures 4a,b) to the U. S. Census Bureau projections (34, 35). The model predicted smoking and ST product prevalence were comparable to the estimated adult smoking prevalence from NHIS (2000–2018) and adult ST product prevalence (2012–2018) (Figure 4c).

### Estimating the impact of introducing *on!* nicotine pouches on the population as a whole

**Figure 5:**
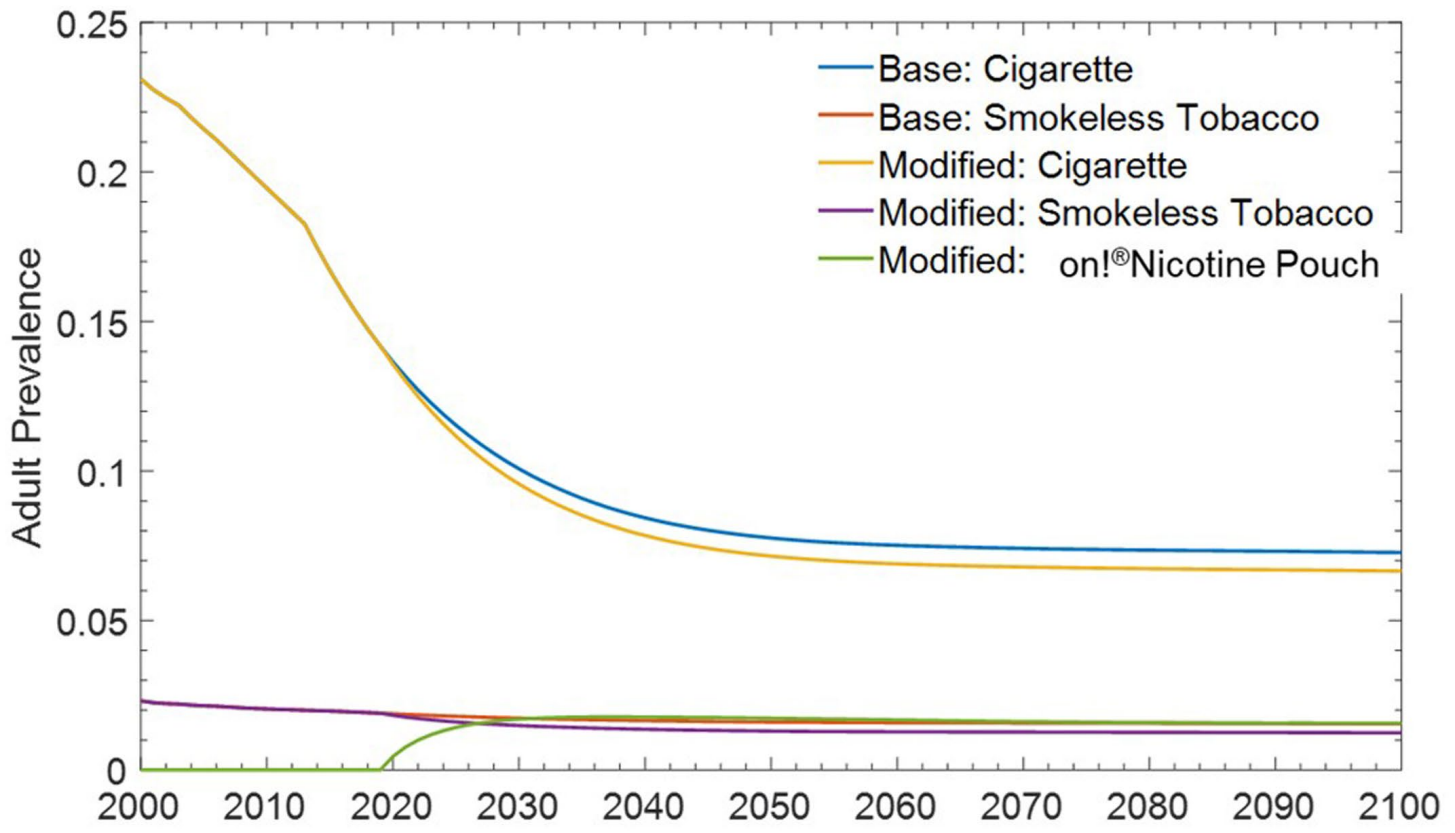
Change in prevalence between base case and modified case for cigarettes, ST products, and *on!* nicotine pouches.

**Figure 6:**
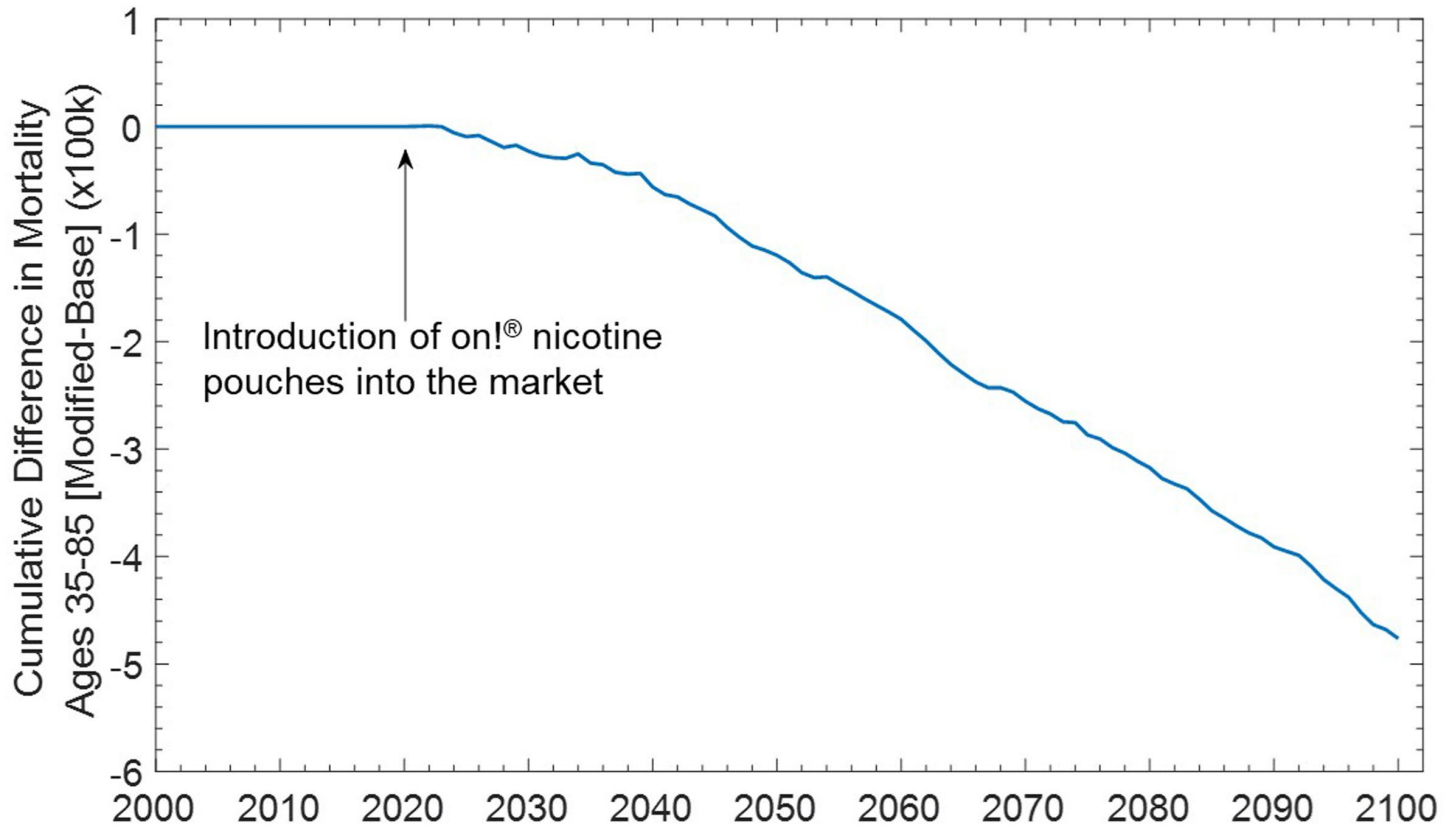
Projected reductions in all-cause mortality upon market authorization of on! Nicotine pouches. The figure shows that any cumulative differences between base case and modified case approaching greater than zero indicate an increase in mortality and any differences <0 indicate reduction in premature deaths. If the predicted cumulative differences equal zero, there are no changes to mortality.

The model predicts that a net benefit to the population as a whole can be expected upon the introduction of *on!* nicotine pouches in the market. The model predicts that smoking prevalence and ST product prevalence would decline by 0.62 and 0.3% respectively, accompanied by a 1.6% increase in prevalence of *on!* nicotine pouches, by the year 2,100 (Figure 5). This decline in prevalence can translate into a reduction in all-cause mortality between the Base and Modified Case scenarios, with the prevention of ~476,000 premature deaths by the year 2,100 (Figure 6).

**Figure 7:**
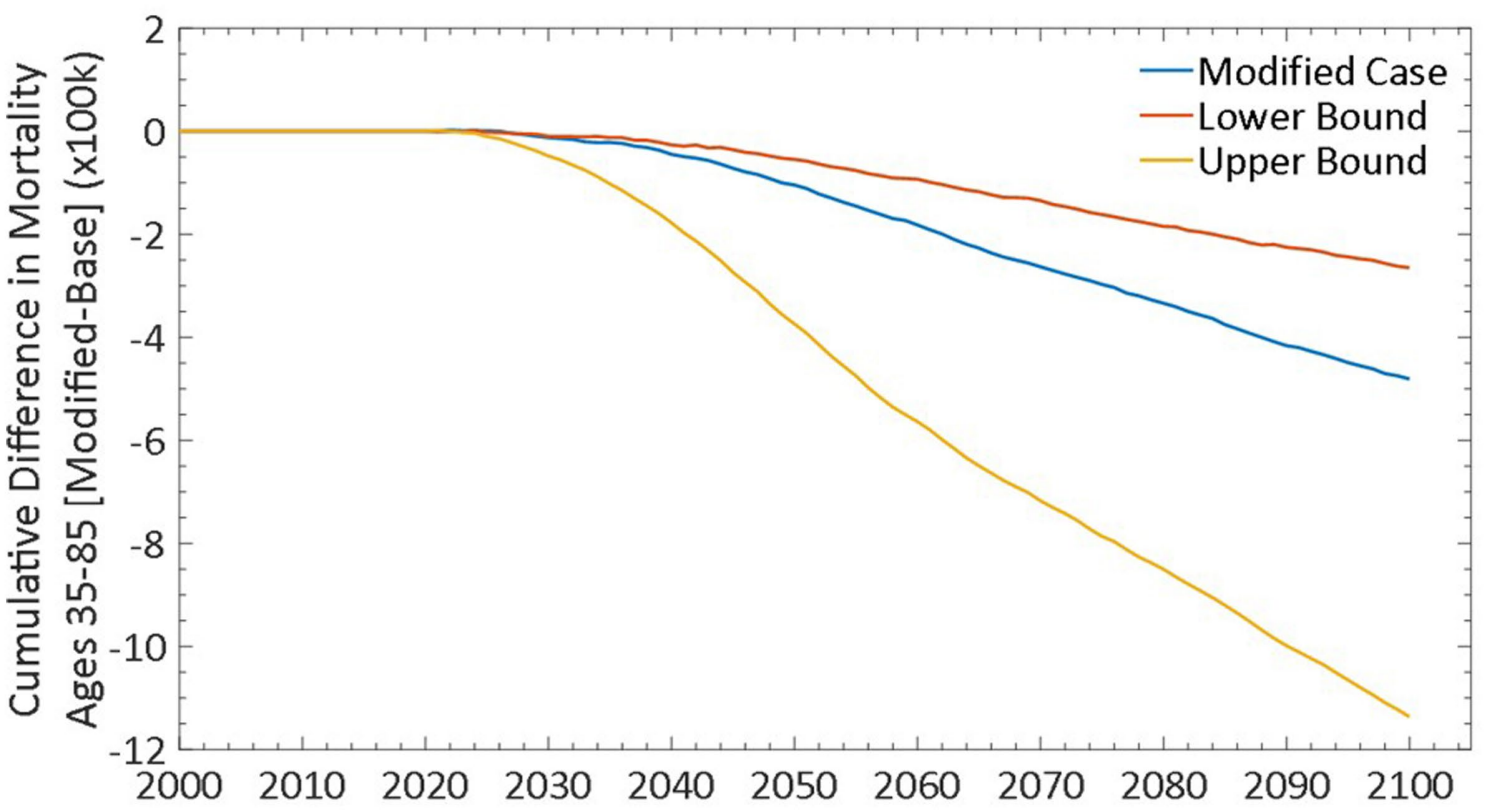
Cumulative differences in all-cause mortality with sensitivity analyses.

Lower and Upper bound scenarios using the lower and upper limits of transition probabilities previously described for *on!* nicotine pouches, while holding all other input transition probabilities constant. We estimated that even the more restrictive criteria in the Lower Bound scenario did not offset the benefit. As such, 240,000 premature deaths would be prevented by 2,100 (Figure 7) with reductions in cigarette prevalence of 0.33% and ST product prevalence of 0.16% and an increase in *on!* nicotine pouch prevalence of 1.3%. (Figure 5) Conversely, under less restrictive criteria in the Upper Bound scenario, we estimated the prevention of 1.1 million premature deaths by 2,100 (Figure 7) with larger reductions in cigarette and ST product prevalence of 1.38 and 0.25%, respectively, and an increase in *on!* nicotine pouch prevalence of 2.58%.

### Sensitivity analysis

To better understand the risk–benefit balance at the population level for the scenario involving the introduction of *on!* nicotine pouches into the U. S. market, we conducted a range of sensitivity analyses by comparing the outcomes from a number of Modified Case scenarios.

A tipping point analysis was conducted by varying only the initiation rates of *on!* nicotine pouch use among individuals who had never used tobacco, while keeping all other transition rates identical to those in the Modified Case scenario. We applied extreme, hypothetical increases up to 4,800% to the initiation rates to determine the point at which the net public health benefit would be neutralized. The results indicated that, under this highly unlikely scenario, the tipping point occurs at a 3,500% increase in initiation rates (Table 3). At this threshold, the cumulative difference in all-cause mortality compared to the Modified Case becomes zero, meaning no net population benefit would remain.

**Table 3:** Impact of increasing initiation rates of *on!* nicotine pouches.

Percentage change in initiation rate	Cumulative premature deaths prevented in 2100
0% (Modified case)	476,000
100%	463,000
400%	409,000
800%	370,000
2,400%	149,000
4,800%	−171,000

**Figure 8:**
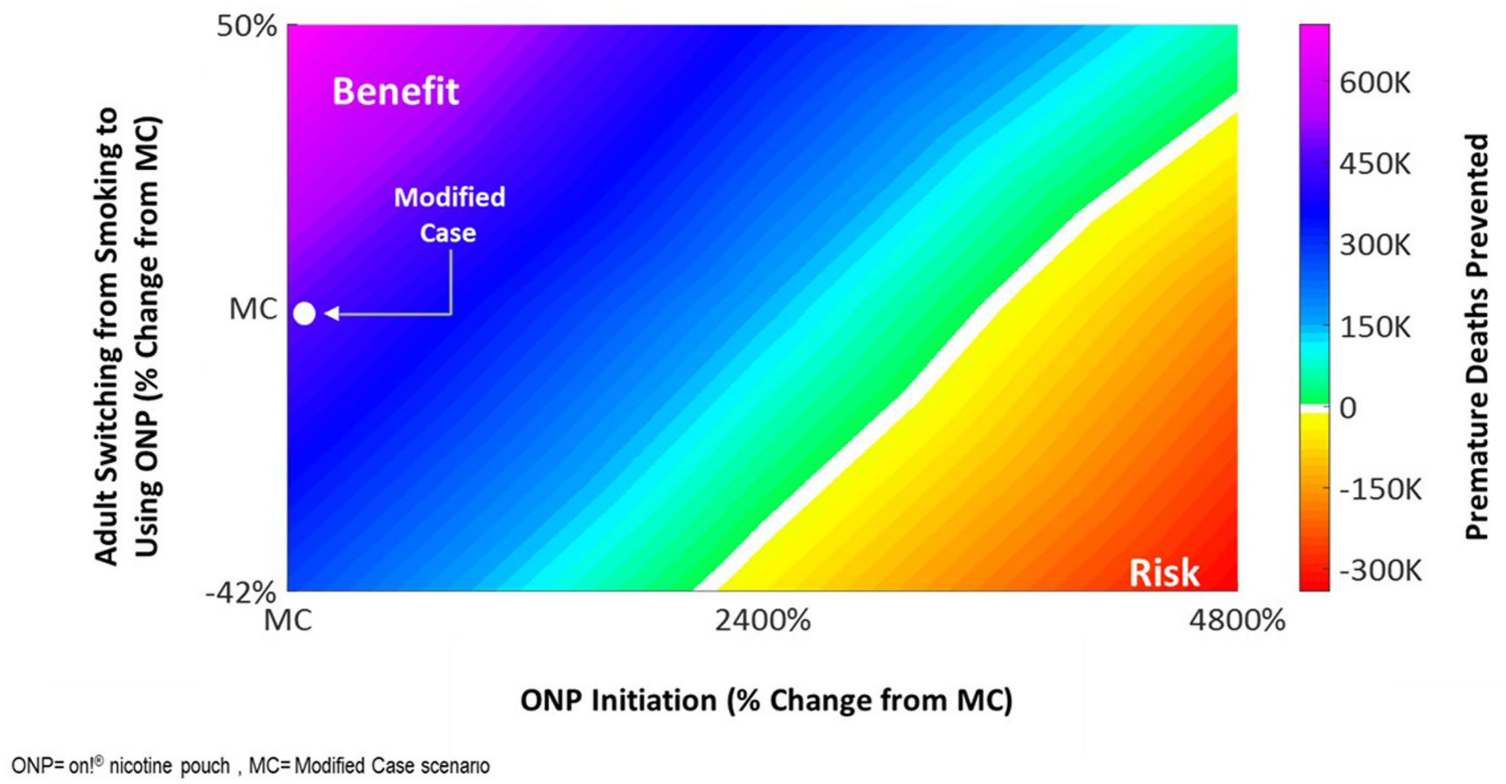
Impact of concurrently varying initiation and switching rates from cigarettes to on!^®^ Nicotine pouches.

Bivariate sensitivity analysis provides a valuable approach to evaluating risk–benefit trade-offs by simultaneously varying two variables and assessing their combined impact on population-level outcomes. We concurrently varied the transition probabilities for a key risk variable: initiation with nicotine pouches among nonusers, including youth and a key beneficial variable: adult users switching from smoking or ST product, holding all other transition probabilities constant. We estimate that a hypothetical increase in initiation rates by 4,800% (from 0.02 to 1.0%) is offset by the relatively small increase in switching rate of 50% (from 0.076 to 1.4%) (Figure 8).

## Discussion

This study employs a validated three-product-based agent-based model to evaluate the public health impact of *on!* nicotine pouches over an 80-year simulation period (2020–2,100). Since adults who smoke and/or use ST products are likely users of these products, the model integrates transition and mortality sub-models and is contextualized upon the introduction of these nicotine pouches within a present-day market. Transitioning to *on!* nicotine pouches are projected to prevent nearly half a million premature deaths by 2,100, while also contributing to a decline in the prevalence of cigarettes and ST products.

Computational models, such as the ALCS ABM, offer a unique ability to project likely impacts at the population level by incorporating variations in key input parameters related to potential risk (e.g., initiation among non-users, particularly youth) and benefits (e.g., adult switching). The modeling approach allows for dialing up or down, one or both of these parameters, through sensitivity analyses. We observe favorable public health outcomes, even under an unlikely and extreme hypothetical scenario where youth initiation rates increase by up to 3,500% and adult switching rates remain constant. Such analyses also allow for the determination of a tipping point wherein youth initiation increasing by > 4,800% may result in a negative public health outcome if adult switching rates remain constant. However, this can be offset to a favorable outcome if adult switching rates increase by a modest 50%. These analyses are critical, acting as an early warning system—a veritable “canary in the coal mine” enabling manufacturers and regulators to closely monitor trends in youth initiation and proactively implement measures to mitigate risks before they lead to negative consequences. These analyses also facilitate the evaluation of risk changes, such as higher initiation rates, within the context of benefits like adult tobacco consumer switching.

While previous studies have examined the public health implications of market authorization for e-cigarettes, HTPs, or hypothetical smoke-free tobacco products (1–5, 7, 10, 11, 17, 19–21, 36) utilizing a two-product model, we present a more robust approach with a three-product model. The ABM reflects the impact on the population most likely to use the *on!* nicotine pouches, adults who smoke and those who use ST products exclusively or adults who dual use ST product with cigarettes. There is a reason for the disproportionate impacts with initiation of pouches in our bivariate sensitivity analysis. This is because there is a much larger reduction in risk when switching from cigarettes to *on!* nicotine pouch product use (95% reduction in risk relative to smoking) relative to initiation with *on!* nicotine pouch product use among never tobacco users (5% increase in risk relative to smoking). A public health benefit is observed as a result of adults who smoke cigarettes switching to exclusive use of *on!* nicotine pouches outweighing the impact of initiation among never tobacco users.

This analysis leverages robust, validated, and comprehensive public datasets relevant to U. S. populations, offering a novel perspective on tobacco use dynamics.

A systems dynamics model involving three tobacco products; HTPs, e-cigarettes, and cigarettes was developed to examine the impact of HTPs on public health in Italy (6). This study relied on age and gender stratification, as well as extrapolated transition datasets from the US and Japan, due to the limited availability of public data on product transitions (6). While such studies have provided valuable insights into the potential positive public health impact of smoke-free products, the use of diverse datasets for transition modeling has resulted in inconsistent definitions of product use scenarios.

Three-product ABM approaches demand extensive amounts of diverse publicly available health data, which are often limited in availability or accessibility and rely on broad assumptions (4–7, 17). A key strength of this study is its use of a robust array of established and validated data sources, including U. S. census data, literature reviews, U. S. survey data, and databases on U. S. mortality and transition-specific metrics. This study assures a rigorous and comprehensive three-product population health model framework by focusing on data specific to the U. S. population and eliminating potential inconsistencies from extrapolating from disparate international data sets and sources.

While our study provides valuable insights, it is important to acknowledge several limitations and assumptions inherent in the ALCS ABM model. The model assumes that all agents act independently, without inter-agent interactions, and that new agents share the same attributes as the initial population an oversimplification compared to real-world dynamics. There is also an underlying assumption of no changes to current tobacco use prevalence despite marketing of other new tobacco products. Dynamic market forces, changes in the regulatory environments, and policies over the 80 year period can impact the tobacco retail and transition landscape which are difficult to account for, in the current model. Some anticipated regulatory policies may include new product standards. Consequently, the model may require revision as new evidence emerges, serving as a component of an ongoing post-marketing surveillance program. Another limitation of the ABM is that it focuses solely on age and gender and did not include other well-established determinants of smoking prevalence (37–39). Additionally, given that the model is based on transitions for three-products, we limited the number of transition use states for the users and nonusers to avoid creating a complex model with a large number of transition probabilities which would be based on assumptions without empirical evidence. Nonetheless, we include 46 transitions states, with product specific data from the two studies (AUS and PBI Study) to estimate transitions between current- and new- product use states. While these may reasonably offset some of the limitations, the AUS participants represent only a subset of the population of adults who smoke, which may limit the generalizability of our findings. We have attempted to overcome this limitation by including some transition probabilities based on PATH Wave 1 and Wave 2, a nationally representative survey. Finally, given the rapidly evolving tobacco product landscape, limiting the Base Case to cigarettes and ST products may provide only a partial view of transitions to other tobacco products. Nevertheless, expanding the Base Case to encompass additional products would introduce substantial methodological complexity and necessitate robust, high-quality transition rate estimates for a markedly larger set of potential transitions, requirements that exceed the practical limits of a three-product modeling framework. As previous studies have noted, these models do not make any claims about the future prevalence of other tobacco products not specified in the model and any resultant changes in mortality (6). It is important to remember that current model predictions are firmly rooted in the continuation of current use behaviors and prevalence trends (6).

## Conclusion

In conclusion, the validated three-product ALCS ABM demonstrates that the market authorization of *on!* nicotine pouch products can result in a net public health benefit to the U. S. population. The model predicted prevention of 476,000 premature deaths, with a decline in smoking and ST product prevalence of 0.62 and 0.30%, respectively, over the 2020–2,100 timeframe. Sensitivity analyses, which considered the lower and upper bound of the Modified Case scenario, estimated preventable premature deaths ranging from 240,000 and 1.1 million. Moreover, the tipping point analysis suggests that a > 4,800% increase in the risk of youth initiation could likely offset the benefit, only if the adult switching rates remained constant. On the other hand, a modest increase of 50% adult switching rate can offset the hypothetical increase in youth initiation by 4,800%. These findings underscore a net benefit at the population level which outweighs the potential risks, supporting the harm reduction potential of *on!* nicotine pouches.

## Data Availability

The original contributions presented in the study are included in the article/supplementary material, further inquiries can be directed to the corresponding author.

## Footnotes

1 Since this model was developed and validated by scientists at Altria Client Services LLC (ALCS), we abbreviate this model as ALCS ABM to differentiate from other ABMs.

2 ^®^*on!* is a registered trademark of Helix and after first mention will be subsequently referenced throughout this manuscript as *on!* nicotine pouches or *on!* NP.

3 The response to the first question in this outcome measure is measured on a 6-point scale, ranging from 1 to 6, with 1-strongly disagree; 2- disagree, 3-somewhat disagree; 4- somewhat agree; 5-agree; 6- strongly agree. The response to the two subsequent questions are also measured on a 6-point scale, ranging from 1 to 6, with 1-definitely not; 2- very unlikely; 3-somewhat unlikely; 4- somewhat likely; 5-very likely; 6- definitely. A composite score of intention to try is calculated by taking the average of three responses to the questions. The “Likelihood” to try the *on!* NP products is defined as having a positive intention to try the products (having a composite score ≥ 3.5) and indicating a “yes” to purchase intent for the products.

## Author’s note

HC was a former employee of Altria Client Services LLC.
